# Dual Mode NO_x_ Sensor: Measuring Both the Accumulated Amount and Instantaneous Level at Low Concentrations

**DOI:** 10.3390/s120302831

**Published:** 2012-03-01

**Authors:** Andrea Groß, Gregor Beulertz, Isabella Marr, David J. Kubinski, Jaco H. Visser, Ralf Moos

**Affiliations:** 1 Department of Functional Materials, Bayreuth Engine Research Center (BERC), University of Bayreuth, 95440 Bayreuth, Germany; 2 Ford Research and Advanced Engineering, Dearborn, MI 48124, USA; E-Mails: dkubinsk@ford.com (D.J.K.); jvisser@ford.com (J.H.V.)

**Keywords:** dosimeter, signal derivative, sensitivity NO/NO_2_, operation temperature, measurement range adaption, response rise/recovery time, exhaust gas sensor

## Abstract

The accumulating-type (or integrating-type) NO_x_ sensor principle offers two operation modes to measure low levels of NO_x_: The direct signal gives the total amount dosed over a time interval and its derivative the instantaneous concentration. With a linear sensor response, no baseline drift, and both response times and recovery times in the range of the gas exchange time of the test bench (5 to 7 s), the integrating sensor is well suited to reliably detect low levels of NO_x_. Experimental results are presented demonstrating the sensor’s integrating properties for the total amount detection and its sensitivity to both NO and to NO_2_. We also show the correlation between the derivative of the sensor signal and the known gas concentration. The long-term detection of NO_x_ in the sub-ppm range (e.g., for air quality measurements) is discussed. Additionally, a self-adaption of the measurement range taking advantage of the temperature dependency of the sensitivity is addressed.

## 1. Introduction

Due to the tightening emission and safety regulations, the detection of harmful or toxic gases has become a large field of research in the past decades. Resistive or impedimetric solid state gas sensors that detect the actual concentration of a target gas are widely investigated. Usually, n-type semiconducting metal oxides are used that change their resistivity more or less selectively as a function of an analyte concentration. Numerous reviews on gas sensor devices of the so-called Taguchi type are available [1–4]. For NO_x_ detection, the state-of-the-art metal oxide gas sensors are mostly based on WO_3_ [5], SnO_2_, TiO_2_ [6], or In_2_O_3_ [7]. The resistance of these sensors is continuously measured to obtain the actual concentration of the gaseous analyte, *c*_NOx_. Important measurement criteria for such sensors are—besides stability and cross-sensitivity to other varying gases—the sensitivity, the measurement range, and the sensor response time. In the automotive field, a concentration-detecting NO_x_ sensor based on two pumping cells is applied to control the proper function of the NO_x_ reduction catalysts and for On-board diagnostics (OBD). As reported in [8], the sensing performance of this Al_2_O_3_/ZrO_2_ based sensor in the second generation was highly improved by enhancements in the sensor design and control module, but the zero level value has still a uncertainty limiting the reliable detection in the low ppm range.

For the application in the automotive or industrial exhaust gas stream as well as for the air quality management, the mean values over a longer measurement period are of interest. For the latter, the requested NO_2_ thresholds are given e.g., from the EU immission legislation [9] and the German air quality standards (BImSchV [10]) as

- the annual mean value (40 μg/m^3^),
- the 1-hour value (200 μg/m^3^, not to be exceeded more than 18 times a calendar year) and
- the alert value (400 μg/m^3^ measured over three consecutive hours).

The 1-hour limit of 200 μg/m^3^ corresponds to a mean NO_2_ concentration of 0.1 ppm. The detection of low concentrations of analyte gas over a long measurement period, like for instance for air quality monitoring purposes, is challenging with such types of concentration-detecting sensors described above. Noisy signals and the sometimes insufficient long-term stability of the sensor response at 0 ppm (baseline drift) [8,11,12] reduce the ability of these resistive or amperometric sensors to accurately determine the presence of low NO_x_ levels. In addition, typical semiconductor gas sensors show an acceptable response rise time when the analyte, here NO or NO_2_, is added to a test gas. However, when the analyte is turned off, often only a slower recovery time (response fall time) is observed, even in the case of metal oxide sensitive layers with very high surface areas interacting with the gas phase [1,5,13,14]. Reasons for that may be a slow desorption of the strongly sorbed analyte NO_2_, and/or the non-linear sensor response of metal oxide gas sensors that also leads to a non-symmetry in the sensor’s response rise and recovery times. For all these reasons, it may be insufficient to determine the time averaged emission by an integration over time of the response of a concentration-detecting sensor.

To overcome this problem and to be able to detect reliably even small levels of an analyte gas over a longer period, the integrating (also called accumulating) sensing principle has been developed for NO_x_ detection [15–17]. Analyte gas molecules that hit a sensitive layer are accumulated (*i.e.*, sorbed in any way) in this layer. For a useful accumulative-type sensing material, at least one easy-to-detect physical material property should change linearly with the amount of sorbed analyte. For sufficient time intervals, the accuracy of an integrating sensor at low analyte concentration is increased compared to state-of-the-art sensors due to the accumulative properties of the sensitive layer. Even small analyte concentrations will accumulate and contribute to the sensor response. With such a sensor, the mathematical integration of a noisy concentration correlating sensor signal is not needed to determine the time-integrated amount. The ability of detecting the total amount of an analyte by chemical accumulation like a dosimeter or an active sampler, makes this sensor principle suitable for long-term monitoring purposes. In such applications, one is interested in the total amount of analyte that occurs per time period at the sensor (sometimes also called dose). However, also the peak concentrations are sometimes of interest.

It will be shown for an accumulation-type NO_x_ sensor that both quantities, *i.e.*, the dosed amount and instantaneous concentration, can be detected with just one sensor device. The response of such a senor correlates with the total amount of NO_x_ gas, *A*_NOx_, dosed over a time interval and the concentration, *c*_gas_, at any given moment can be obtained from the derivative of this response with respect to time. Furthermore, we find that the sensor response is linear even in the very low ppm region and responds quickly to rapidly changing NO_x_ concentrations. Both response and recovery times are about 5 to 7 s which is in the range of the gas exchange time of the test bench upstream of the sensitive device of 6 s. The measurement range can be self-adapted taking advantage of the temperature dependency of the sensitivity. This makes the sensor suitable for many applications. Cross-sensitivities are shortly addressed.

## 2. Fundamentals

### 2.1. Fundamentals of the Accumulating Sensing Principle

**Figure 1.**
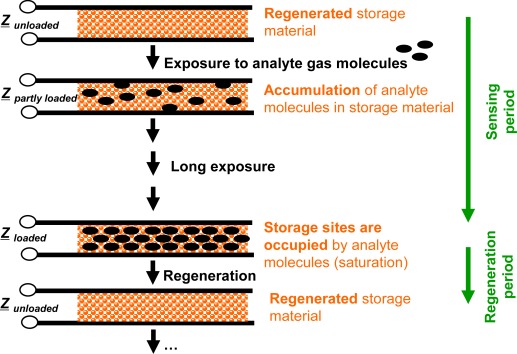
Operation scheme of accumulating-type gas sensors: Long sensing periods (successive accumulation of the analyte molecules in the sensitive layer) alternate with short regeneration intervals (recovery of storage capacity to avoid saturation effects). Here, the complex impedance, *Z*, is measured.

Compared to classical gas sensors, the accumulating gas sensing principle is different. Basic elements of accumulating type sensors are analyte gas storing materials applied as sensitive layers. Their material composition changes successively during gas exposure as the accumulation of the analyte molecules occurs in form of material transformations at the storage sites, *i.e.*, in form of chemical reactions. Typically, such material transformations are reflected by the materials’ electrical properties [15,16,18–23], in particular by their complex impedances, *Z*, by the materials’ mass [24,25] or by their optical properties [26,27]. All these properties may serve as measurands. Therefore, the sensor response is expected to correlate with the total amount (dose), *A*_gas_, of analyte molecules being present in the gas stream over a time interval. Since the storage capacity is limited, the storage sites need to be regenerated as saturation effects deteriorate the integrating properties. These processes are illustrated in Figure 1 for the case of an impedimetric integrating-type gas sensor. Starting with the freshly regenerated material, the analyte gas molecules can be accumulated in the sensitive layer. After this accumulation phase, the storage sites are occupied and the operation mode needs to be shifted to the regenerating conditions to recover the original storage capacity. In the ideal case, long sensing periods alternate with short regeneration intervals.

**Figure 2.**
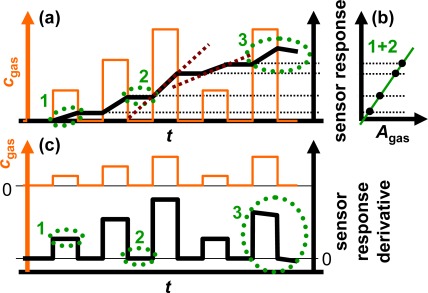
Fundamentals of the sensor response of an ideal accumulating-type gas sensor. (**a**) Increasing sensor signal on the time scale during cyclic gas exposure due to accumulation. (**b**) Resulting characteristic line: correlation with the total amount *A*_gas_. (**c**) Curve of the signal derivative: correlation with the actual concentration *c*_gas_.

The sensor response of an ideal integrating sensor during cyclic analyte gas exposure with the analyte gas concentration *c*_gas_ is illustrated in Figure 2(a). On the time scale, the sensor response increases in the presence of the analyte gas due to the accumulation of analyte molecules [linearity (1)] but remains constant in the absence of the analyte in the gas stream since the total amount of the stored analyte is not changing [holding capability (2)]. As an undesired effect, in the highly loaded state, the sensitivity may decrease and some of the formerly stored analyte molecules might desorb at 0 ppm analyte gas [saturation effects (3)]. The corresponding characteristic line in Figure 2(b) is obtained by correlating the sensor response with the amount of analyte gas, *A*_gas_. *A*_gas_ here is defined as:

(1) $$A_{\mathrm{gas}}\;(t)=\int c_{\mathrm{gas}}\;(t)\;d\;t$$

resulting in the total exposed amount of analyte during the sensing period in the unit ppm·s. Due to the analyte gas accumulation of the sensitive layer, the characteristic line in Figure 2(b) gives a linear correlation between the sensor response and *A*_gas_, which is not affected by the analyte gas concentration *c*_gas_. Therefore, the total amount of analyte gas (the dose) since the last regeneration period is detected directly with the integrating sensor due to the accumulating properties of the sensitive gas storage material.

Another benefit of the integrating sensing principle is the fact that long-term drifts of the zero level signal (e.g., due to aging of the involved materials)—which often cause inaccuracies in state-of-the-art concentration detecting sensors [8]—are avoided by self-defining the zero value of the sensor response after each regeneration period.

To obtain ideal integrating properties, it is essential that the slope of the sensor signal on the time scale depends linearly on the actual analyte concentration, *c*_gas_, since a doubled concentration results in a doubled amount per time unit. From Figure 2(a) it can be obtained that the slope of the sensor response of the ideal accumulating sensor increases linearly with the concentration of the analyte in the test gas as highlighted by the dashed red lines exemplarily for the third and fourth NO_x_ step. This behavior can be used to determine the actual analyte gas concentration, *c*_gas_. As sketched in Figure 2(c), the timely derivative of the response of an ideally integrating sensor correlates with the actual concentration of the analyte gas, *c*_gas_, due to the linearity in the presence of the analyte (1) and the holding capabilities at 0 ppm (2). At high loading states, it is expected that saturation effects (3) lead to deviations from the ideal behavior.

Utilizing the sensor response of one single accumulating-type sensor and its derivative, one can obtain:

- the total amount of the analyte molecules *A*_gas_ (accumulative properties) from the sensor response and (Figure 2(b))
- the time-resolved actual analyte gas concentration *c*_gas_ (linearity) from the sensor response derivative (Figure 2(c)).

### 2.2. Materials Aspects

The appropriate selection of the material of the sensitive layer is essential for the integrating behavior of the accumulating-type sensor. In general, six main criteria for the material properties have been found to be important for the accumulating measurement principle:

1. Selective sorption: The analyte molecules are accumulated selectively in the sensitive layer, e.g., via a chemical storage process including material conversion. The amount of molecules stored per time unit should be proportional to the actual analyte concentration (linearity). The selectivity ensures negligible cross sensitivities to other gas components.
2. Strength of sorption: The sorption of the analyte molecules needs to be strongly enhanced compared to desorption, ensuring accumulation of the analyte molecules in the sensitive layer and no losses in the absence of analyte in the gas phase (holding capability).
3. A linear relationship between the sorbed analyte amount and the material property, e.g., the conductivity or the impedance, monitored as sensor response (characteristic line) is crucial.
4. The change of the material properties that serve as a measurand must be independent on the analyte loading level.
5. Regeneration: Only under defined conditions that are different from the operation conditions, the sorbed analyte molecules can be released from the storage sites to regenerate the material – otherwise it is a single-use sensor.
6. Measurement range: The storage capacity of the sensitive layer and the rate of sorption need to be adapted to the application requirements.

It is reported that (to some extent) several materials ranging from carbon nanotubes [20] to polymers [24,27], carbonates [25], zeolites [22,23,28] or materials used in passive samplers [29] show accumulative properties, even if it is often not explicitly denoted in the literature as accumulative. In [30], modified diamond surfaces adsorb at room temperature a very thin water film, in which water-soluble gases affect selectively the pH of the film. Since the gases get by far faster solved than dissolved, the pH signal, which can be conveniently measured, behaves like an accumulating sensor.

In our contribution, we focus on carbonate-based lean NO_x_ trap (LNT) materials, known from automotive catalysts, that have been found to change their electrical properties during NO_x_ exposure [18,31] and were therefore tested as NO_x_-sensitive materials working accumulatively like a dosimeter [14,15].

### 2.3. NO_x_ Storage Layer for an Integrating NO_x_ Sensor

Since the long-term reliable detection of low levels of NO_x_ is challenging, the integrating NO_x_ sensor based on a NO_x_ storage layer was developed. Here we present experimental results for such a sensor demonstrating its ability to detect small concentrations of NO and NO_2_ (*i.e.*, 200 ppb for air quality monitoring or 2 to 10 ppm for exhaust gas applications) at different temperatures.

Lean NO_x_ trap (LNT) materials serve as sensitive layers of the presented integrating-type NO_x_ sensor. LNTs were developed as automotive catalyst materials to reduce the NO_x_ emissions in the exhaust of leanly operated engines. They are able to store NO_x_ molecules chemically in the form of nitrates in atmospheres with excess oxygen and to release them in short rich intervals so that the NO_x_ molecules can be reduced to harmless nitrogen [32,33]. The highest NO_x_ conversion occurs in the temperature range from 350 to 400 °C due to kinetic limitations at lower temperatures [32,34,35] and a reduced thermodynamic stability of the formed nitrates leading to decomposition at the higher temperatures [32,33]. Generally, those catalyst formulations consist of [32–37]:

- a porous oxide framework—typically stabilized alumina—providing a high inner surface area,
- alkaline (earth) metal oxides or carbonates “MCO_3_” (e.g., of potassium or barium) being able to store NO_2_ molecules in the form of nitrites or nitrates “M(NO_3_)_2_”
  (2) $${\mathrm{MCO}}_{3}+2{\mathrm{NO}}_{2}+1/2\;O_{2}↔M{\left({\mathrm{NO}}_{3}\right)}_{2}+{\mathrm{CO}}_{2}$$
- oxygen storage components like zirconia stabilized ceria and
- finely dispersed precious metal particles to catalyze oxidation and reduction reactions, e.g.,
  (3) $$\mathrm{NO}+1/2\;O_{2}↔{\mathrm{NO}}_{2}$$

The ability to oxidize NO to obtain NO_2_, that can be stored in a second step chemically by converting carbonates to nitrates and to release the formerly stored NO_x_ in rich surroundings or at higher temperatures to recover the storage capacity, make LNTs good candidates as sensitive layers for accumulating total NO_x_ sensors. Jung *et al.* [25] detected the NO_x_ accumulation and release on BaCO_3_, the most popular NO_x_ storage component, at 400 °C gravimetrically by the mass increase when converting the carbonate to nitrate. Since the complex impedance of the LNTs was found to be highly affected by the NO_x_ loading level [21], the presented accumulating total NO_x_ sensor has been developed to detect low levels of NO and NO_2_ by electrical means.

## 3. Experimental Section

**Figure 3.**
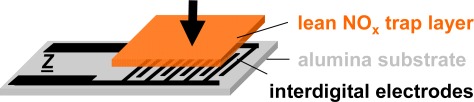
Setup of the accumulating-type NO_x_ sensor.

The setup of the presented resistive type integrating NO_x_ sensor is shown in Figure 3. The sensor devices consist of a 96% pure alumina substrate (630 μm thick) with screen-printed interdigital electrodes (IDE) made from gold (DuPont) or platinum (Heraeus)—the electrode material was found to have no influence on the sensor performance—with an electrode width and spacing of 100 or 50 μm each. The electrode areas (5 × 6 mm) are covered with layers of a potassium-based LNT-material in various thicknesses ranging from about 30–100 μm. The raw material was provided by Johnson Matthey and the composition is described in [38]. The sensors were operated in a quartz tube (25 mm in diameter) located in a heated furnace at 380 °C (furnace temperature) and supplied with various synthetic gas mixtures of 2 L/min. The gas exchange time of the test bench upstream of the sensor (dosing unit, quartz tube) was estimated to be in the range of 6 s.

To avoid polarization effects, the electrical properties were monitored by impedance spectroscopy in the frequency range from 1 Hz to 1 MHz at 1 V (rms). Nyquist spectra in the regenerated and in the NO_x_-loaded state proved that the electrical properties can be described almost perfectly by an *RC* parallel equivalent circuit. The capacitance *C* was found to be only slightly affected by NO_x_, whereas the resistance *R* depended highly on the NO_x_ loading state. To investigate the time-dependent behavior, the complex impedance, *Z*, was recorded every 1 or 2 seconds at 1 kHz and the resistance *R* was calculated from the impedance by:

(4) $$R=|\underline{Z}|\sqrt{1+{\text{tan}}^{2}\;\varphi }$$

with |*Z*| being the absolute value of the complex impedance and *φ* the phase angle. To compare the effect of NO_x_ sorption on different sensor samples, the relative resistance used as sensor response is given in %:

(5) $$|\Delta R|/R_{0}=(R_{0}-R)/R_{0}$$

with *R*_0_ being the resistance in the unloaded state. The sensitivity, *S*, of the sensor is defined as the sensor response change related to the total amount of NO_x_, *A*_NOx_, calculated according to Equation (1) and reflecting the slope in the scheme in Figure 2(b):

(6) $$S=\frac{d(|\Delta R|/R_{0})}{d\;A_{\text{NOx}}}$$

The time derivative of the measured resistance values is correlated with the actual NO_x_ concentration during NO_x_ exposure. The derivative |d*R*/d*t*| is calculated by the absolute value of dividing the resistance change of two subsequent data points by the time (*t*) difference between them, which is usually 1.6 to 1.7 s:

(7) $$|dR/dt|=\left|\frac{R_{n+1}-R_{n}}{t_{n+1}-t_{n}}\right|$$

The response rise time *t_90_* is determined from the measurement curve of |d*R*/d*t*| taking the time difference between the beginning of the increase of the signal due to NO_x_ and reaching 90% of the end value of |d*R*/d*t*| corresponding to the actual concentration. The recovery time is defined as the duration until |d*R*/d*t*| reaches the zero value. For each measurement, the mean values of *t*_90_ and the recovery time concerning the NO_x_ steps besides of the first ones are given.

The integrating behavior has been demonstrated with various sensor devices having slightly different porous sensitive layer geometries and morphologies. These devices, each providing probably a different amount of accessible storage sites, resulted in different absolute resistance values and sensitivities.

As test gas base composition, a humidified gas mixture (50% N_2_ in a water bubbler at room temperature) of 10% O_2_ and 5% CO_2_ in N_2_ was used. The gas composition was mixed using mass flow controllers. A three-way valve was applied to the NO and NO_2_ line. The valve enabled to drain NO_x_ to the vent prior to admixing it to the main gas flow. This avoided pressure pulses resulting in an uncontrolled overdosing of NO_x_ at the beginning of the NO_x_ dosing intervals. The NO and NO_2_ concentrations downstream of the sensor position were verified under steady state conditions with a chemiluminescence detector (Eco Physics CLD 700 EL ht).

## 4. Results and Discussion

In the following six sections, the sensor response and its derivative towards various NO_x_ programs are presented, demonstrating the accumulating properties of this resistive-type integrating sensor under various conditions and the dual mode signal functionalities. The presented NO_x_ runs address the detection of very low levels of NO like 2 ppm for just 25 s during cyclic exposure, the influence of varying NO concentrations, and the comparison of the sensitivity to NO and NO_2_. Additionally shown is a 50 min run with very low levels of NO_2_ (concentration in the sub-ppm range) that was not interrupted by regeneration periods proofing the suitability for air quality measurements. Each plot is arranged as in the scheme in Figure 2. It consists: (a) of the sensor response |Δ*R*|/*R*_0_ during cyclic NO_x_ dosing; (b) of the corresponding characteristic line correlating |Δ*R*|/*R*_0_ with the total amount of NO_x_, *A*_NOx,_ and (c) of the curve of the resistance derivative |d*R*/d*t*| mirroring the actual NO_x_ concentration, *c*_NOx_. As a reference, *c*_NOx_ calculated with data of the mass flow controller outputs is shown as well. The demonstration of the performance of the integrating NO_x_ sensor and its benefits is complemented in a fifth section with results on the temperature dependency of the sensor response, which can be employed to self-adapt the measurement range to the requirements. Since the selectivity of gas sensors to the analyte strongly affects accuracy, cross-sensitivities to other present gases will be discussed in the last section.

### 4.1. Detection of Low Levels of NO

**Figure 4.**
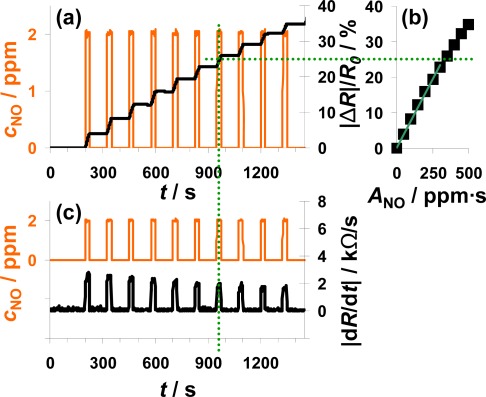
Sensor response on 10 pulses of 2 ppm NO each for 25 s alternating with 0 ppm for 100 s.

In Figure 4, the sensor response towards a pulsed addition of 2 ppm NO to the lean gas mixture is shown. Starting at 200 s, 2 ppm NO for 25 s alternate with 0 ppm NO for 100 s. The orange line (left axis) is the NO concentration as calculated from the mass flow controller outputs. The constant resistance value before the first NO step was used as *R*_0_ for the calculation of the sensor response |Δ*R*|/*R*_0_ according to Equation (5). As can be seen in Figure 4(a), |Δ*R*|/*R*_0_ increases in the presence of 2 ppm NO due to an increased conductivity of the NO_x_ trap layer (*R* < *R*_0_). The slope appears to be almost constant during each NO pulse period. In the absence of NO, |Δ*R*|/*R*_0_ remains constant. Both, the linear increase of the sensor response in the presence of NO and the constant sensor response at 0 ppm NO confirm the integrating behavior of the sensor.

The characteristic line of the integrating NO_x_ sensor correlates the sensor response |Δ*R*|/*R*_0_ with the total amount of NO_x_, *A*_NOx_, calculated from Equation (1). The characteristic line was extracted from the measurement data after each NO step (Figure 4(a)) and plotted in Figure 4(b). The correlation between |Δ*R*|/*R*_0_ and *A*_NOx_ is linear up to a sensor response of about 25% and the sensitivity, *S*, (Equation (6)) in this measurement range is about 0.078%/ppm·s for this 100 μm Pt-IDE sensor. At higher loading states, the characteristic line gets nonlinear and the sensitivity decreases (denoted by the dotted line). As described above, the slope of the sensor response of an integrating gas sensor correlates with the actual concentration, *c*_gas_, at a constant gas flow rate. To prove this correlation, the curves of the absolute value of the derivative of the resistance |d*R*/d*t*| and of the added NO concentration, *c*_NO_, are compared in Figure 4(c). The resistance changes by about 2.5 kΩ/s in the presence of 2 ppm NO in the lowly loaded state. This value decreases slightly with time due to saturation effects. In the case of 0 ppm NO, the derivative is zero (holding capabilities) independent on the baseline-resistance.

The measurement results shown in Figure 4 clearly prove that the described NO_x_ sensor based on a LNT-sensitive layer works like an integrating sensor. In addition, the measurement results clarify that it is well adapted to detect even very small NO_x_ levels like 2 ppm NO for just 25 s with a high sensitivity (here: 0.078%/ppm·s NO). In contrast to classical gas sensors, the response time and the recovery time are identical and very fast in the range of 4 to 5 s. Since this is approximately the gas exchange time of the test setup, it is an indication that the sensor might respond even faster. The correlation between the signal derivative and the instantaneous analyte concentration is obvious. It should be noted here that the use of the time derivate of the sensor response to increase the speed of its response is not novel. It has been successfully applied for instance for the detection of ozone with phthalocyanine thin films [39]. However, in these cases, the sensor materials do not show an integrating behavior, but they do respond slowly to the changing analyte concentration. In other words, they do not collect the offered analyte molecules and do therefore also desorb the analytes after turning off the analyte.

### 4.2. Effect of Different NO Concentrations

**Figure 5.**
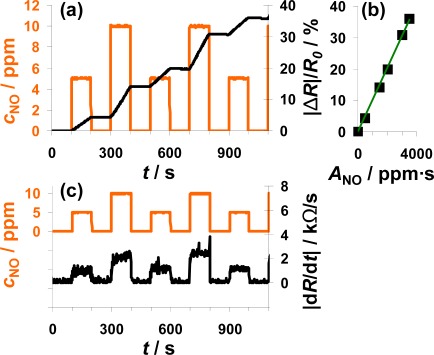
Sensor response on pulses of three times 5 ppm and two times 10 ppm NO for 100 s each.

Another interesting point is to investigate whether the sensor also works as an integrator if the NO concentration varies. The sensor response to alternating 0, 5, and 10 ppm NO for 100 s each is plotted in Figure 5. Again, the sensor response increases during NO exposure but remains constant in the absence of the analyte (Figure 5(a)). In the case of 10 ppm NO, |Δ*R*|/*R*_0_ increases faster than in the presence of 5 ppm—the slope seems to be doubled. By calculating *A*_NO_ from Equation (1), the doubled concentration is taken into account. Looking at the characteristic line in Figure 5(b), it becomes obvious that the measurement points form a straight line if plotted *versus A*_NO_. This means that the characteristic line in Figure 5(b) is independent on the actual concentration and that the sensor works as an NO-accumulating device, detecting the total amount of NO in the low ppm range. The sensitivity of this sensor device (100 μm Pt-IDE) is 0.010%/ppm·s NO. The sensitivity obtained in this measurement is much smaller compared to the sensitivity reported in the previous section (0.078%/ppm·s NO) in Figure 4. The measurement results presented were obtained with different sensor samples that were coated by screen-printing or dipping in an aqueous solution of the LNT-material resulting in sensitive layers with varies thicknesses and morphologies. In further tests, it was found that the sensitivity to NO_x_ varies with the thickness of the sensitive layer, which can be explained by the dependency of the number of accessible storage sites on the volume and the morphology of the sensitive layer influencing the diffusion of NO_x_ into the storage material and therefore the rate of storage. In Figure 5(c), |d*R*/d*t*| is plotted in parallel to *c*_NO_ and follows it. In the presence of 5 ppm NO, the resistance changes by about 1.25 kΩ/s, in 10 ppm it is about 2.5 kΩ/s, which demonstrates once more the linearity. Again, the sensor signal responds and recovers very fast (5 to 6 s) with only a slight dependency on the actual NO concentration.

From a chemical reaction point of view, the results indicate that in the low loading state and in the case of low concentrations, the sensitive layer is able to incorporate always a constant fraction of the incoming NO molecules, *i.e.*, the reaction rate of the rate determining reaction (either Equation (2) or Equation (3)) is constant. These results show further that the amount of NO flowing in a tube can be measured independently of the actual NO concentration in the low ppm-range and that the actual concentration can be determined by looking on the derivative of the sensor response.

### 4.3. Sensitivity to NO and NO_2_

To obtain a total NO_x_ sensor, it is essential that the sensor responds to NO the same way as it does to NO_2_. This means for an integrating NO_x_ sensor that the linearity as well as the holding capabilities should be equal for both NO and NO_2_.

**Figure 6.**
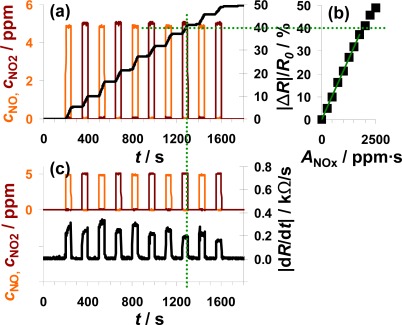
Sensor response on alternating pulses of 5 ppm NO (orange) and 5 ppm NO_2_ (red) for 50 s.

NO and NO_2_ were added alternately to the lean base gas (Figure 6). Each step consisted of 5 ppm NO or 5 ppm NO_2_ lasting 50 s which were separated by 100 s intervals of 0 ppm NO_x_. Figure 6(a) shows the sensor response as a function of time together with the dosed analyte gas concentrations. NO_x_ dosing started with NO (orange line) which was later followed by a NO_2_ pulse (dark red line), with this cycle repeated five times. The sensor responds to NO in an integrating manner as already discussed in Figures 4 and 5. In the presence of NO_2_, |Δ*R*|/*R*_0_ increases with a constant slope and after switching off the NO_2_ flow, |Δ*R*|/*R*_0_ remains constant. Comparing the course of |Δ*R*|/*R*_0_ during NO and NO_2_ exposure, the sensor works accumulative for NO_x_: both linearity and holding abilities seem not to be affected whether NO or NO_2_ is added to the gas. This observation is verified by plotting the measurement data as a characteristic line similar to that shown in the previous two figures. The correlation between |Δ*R*|/*R*_0_ and *A*_NOx_, with *A*_NOx_ being the sum of *A*_NO_ and *A*_NO2_, gives a linear line up to a sensor response of about 40% with a sensitivity of 0.021%/ppm·s (50 μm Au-IDE). This demonstrates graphically the relative independency of the sensor response on the NO/NO_2_-ratio. Like the sensitivity, the difference in the range of linearity varies between the used samples. Having a closer look on the timely derivative in Figure 6(c), the correlation between |d*R*/d*t*| and the NO_x_ composition can be seen in much more detail. In the presence of 5 ppm NO, the resistance decreases about 0.30 kΩ/s compared to about 0.25 kΩ/s at 5 ppm NO_2_. Again, we also see in Figure 6(c) the trend of a smaller sensor response with increasing NO_x_ loading, when the saturation becomes noticeable. As in the previous measurements, the sensor response time is in the range of the gas exchange time of the system (6 s) and is not influenced by the composition of the dosed NO_x_. Unlike before, the curve of |d*R*/d*t*| recovers after a NO_2_ step in 12 s whereas it takes 17 s after NO exposure.

The data in Figure 6 demonstrate the ability of the presented integrating sensor to detect low levels of NO and NO_2_ with almost the same sensitivity. The integrating properties seem not to be affected by the NO_x_ composition and the linearity of the sensor signal as well as the holding capabilities are given for the both analyte gases NO and NO_2_.

According to [40], the storage of NO occurs via the nitrite and the nitrate route, both involving NO oxidation. Since the NO/NO_2_-ratio in automotive exhausts is mainly influenced by temperature, precious metal particles are added to the catalyst formulations to establish the equilibrium reaction of NO and NO_2_ (Equation (3)). If the catalytic activity of the catalyst coating is sufficiently high, the efficiency of the LNT should not be affected by the NO/NO_2_ ratio in the feed gas. The measurement results in Figure 6 demonstrate that the sensor response of the proposed integrating sensor is not affected by the NO_x_ composition and indicate that the oxidizing properties of the sensitive layer are sufficient to accumulate upcoming NO with the same rate as NO_2_.

### 4.4. Long-Term Monitoring of Low Levels of NO_2_

**Figure 7.**
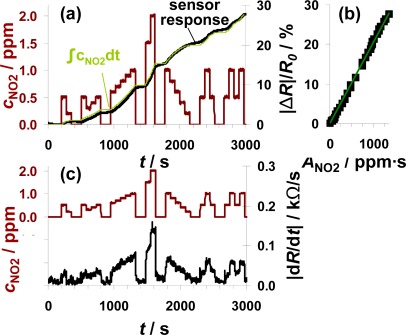
Sensor response towards low levels of NO_2_ from 0.2 to 2 ppm in steps of 75 s each.

To check, whether the sensor can be used for air quality monitoring of NO_2_, the sensor test bench was modified to establish NO_2_ concentrations down to 0.2 ppm. A sensor sample (100 μm Pt-IDE) with an average sensitivity was selected for this test. The total amount of about 1,340 ppm·s NO_2_ was added to the gas stream in several steps of 75 s each with NO_2_ concentrations ranging from 200 ppb to 2 ppm. The red line in Figure 7(a) reflects the curve of the dosed NO_2_ concentrations with the focus on the sub-ppm range. The ppm-values are calculated from the mass flow controller outputs. As in the previous figures, |Δ*R*|/*R*_0_ increases in the presence of NO_2_ demonstrating that the sensor responds to very small levels of NO_2_ in the sub-ppm range (e.g., 200 ppb). The curve of the signal derivative |d*R*/d*t*| in Figure 7(b) was calculated according to Equation (7) and smoothed by a moving average of three values. Even in this sub-ppm range, the slope |d*R*/d*t*| correlates with the actual NO_2_ concentration demonstrating that the integrating properties are only negligibly affected by the actual analyte concentration and that the lowest levels tested can be detected. In periods with 200 ppb NO_2_, the signal changes by about 24 Ω/s. To clarify the amount-detecting properties, the curve of the sensor response |Δ*R*|/*R*_0_ (in black) is directly compared to the expected sensor response in light green in Figure 7(a). This expected curve has been obtained by multiplying the actual fraction of the dosed amount of NO_2_ (integration of the concentration curve) with the end value of the sensor response at 1,340 ppm·s. The measured and the calculated curves coincide very well. Up to about 1,500 s, the curve of the measured sensor response is slightly below the expected curve but exceeds it in the second part. These slight deviations might be due to a slightly increased sensitivity at higher concentrations or by small inaccuracies in the gas dosing system. Interestingly, the sensor response increases in the absence of NO_2_, which cannot be explained by desorption of formerly stored NO_x_. More likely, impreciseness of the modified gas mixing system (non-flushed dead volume in the NO_x_ dosing unit) is responsible for this signal increase. Unfortunately, no additional information could be obtained from the chemiluminescence detector downstream of the sensor, since its resolution is 0.1 ppm. The characteristic line in Figure 7(b) confirms the integrating properties even in the area of small amounts since there is a linear correlation between |Δ*R*|/*R*_0_ and *A*_NO2_ in the measured range up to 1,340 ppm·s with a sensitivity of 0.021%/ppm·s.

The measurement range of 1,340 ppm·s and the NO_2_ concentration of 0.2 ppm are in a good agreement with the European and German air quality standards. As mentioned above, the permitted 1-hour value of NO_2_ is 200 μg/m^3^. This results in an average NO_2_ concentration of about 0.1 ppm. The results in Figure 7 demonstrate that the accumulating type NO_x_ sensor at this stage of development is capable to detect 0.2 ppm of NO_2_ over at least 50 min without regeneration step in between. The permitted level of about 0.1 ppm for 1 h results in a total amount of 360 ppm·s NO_2_. According to the data in Figure 7, one remains in the linear range for almost four hours (3.7 h) if 200 μg/m^3^ (air quality threshold, see introduction) are in the sensor ambient. The permitted annual mean value of 40 μg/m^3^ could be monitored for about 18.5 hours.

### 4.5. Temperature Dependence of the Sensor Performance

Another interesting point is how the temperature dependence of the storage behavior of LNTs (kinetic limitations at low temperatures, decreased nitrate stability at high temperatures) affects the performance of the integrating NO_x_ sensor based on an LNT layer.

**Figure 8.**
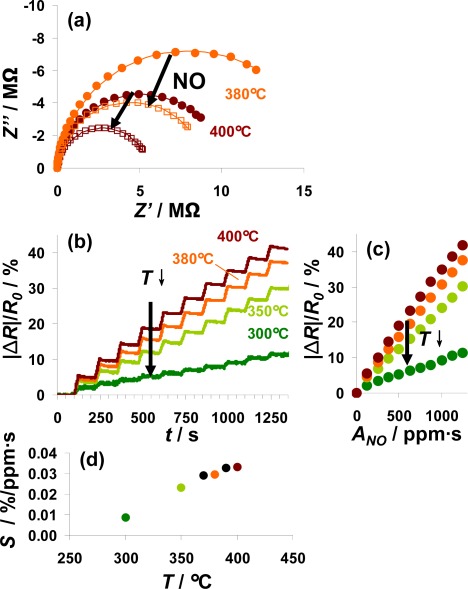
Influence of the temperature on the sensor response to 10 times 5 ppm NO for 25 s.

In Figure 8, the sensor responses of a 100 μm Pt-IDE sensor on NO steps at various temperatures from 300 to 400 °C are compared. For all investigated temperatures, the electrical behavior in the unloaded and partly NO_x_-loaded state can be described by an R||C element. The diameter of the semicircle in the Nyquist-plot, reflecting the resistance, decreased during NO loading as shown in Figure 8(a) for 380 °C (orange) and 400 °C (red line). Data are shown for both prior (solid points) and after (open symbols) exposure to 1,250 ppm·s NO. The conductivity of the sensitive layer in the unloaded state depends on temperature (a detailed analysis gives an activation energy of 0.9 eV). Correspondingly, the resistance of the loaded state also decreases with temperature. The sensor response, |Δ*R*|/*R*_0_, as a function of time is depicted in Figure 8(b) for ten steps of 5 ppm NO for 25 s alternating with 0 ppm for 100 s. This plot shows that the sensor exhibits integrating properties from 300 °C to 400 °C with a reduced sensor response at lower temperatures. Extracting the characteristic lines from these data (Figure 8(c)) demonstrates the linearity of the sensor response up to 400 °C as well as the enhanced sensitivity at higher temperatures. Plotting the sensitivity *S* calculated from the characteristic lines according to Equation (6) as a function of the temperature (Figure 8(d)) points out that the sensitivity to NO correlates nearly linearly with the temperature from 300 to 370 °C. Further rise in the temperature up to 400 °C results in a slightly reduced increase in the sensitivity. As reported in [16], the decreased stability of the formed nitrates at higher temperatures deteriorates the holding abilities of the storage sites and therefore the accumulating behavior of the sensitive layer. At lower temperatures, the kinetics limits the storage rate being reflected by the sensitivity, whereas the linear measurement range is restricted by the stability of the nitrates. Therefore, the sensitivity in the linear detection range increases with temperature whereas this range gets smaller at elevated temperatures. To adapt the measurement range of the integrating NO_x_ sensor to the requirements of the application, the measurement temperature is an effective tool as it is an easy parameter to adjust.

According to the results shown above, a typical measurement procedure would be as follows: The sensor is thermally regenerated at a temperature above 500 °C by decomposing the formed nitrates, and as soon as the sensor is cooled down to its operation temperature, the new value of *R*_0_ is defined. This periodically re-defining of *R*_0_ avoids long-term zero value drifts of the sensor signal, which is a common drawback of state-of-the-art, resistive gas sensors.

The disadvantage is that during regeneration, the sensors remain blind. In other words, the time the sensor needs to cool down from *T* > 500 °C to operation temperature should be as short as possible. With ceramic sensors as shown in Figure 3, typically a thermal time constant in the 10 s ranges is observed [41]. However, reducing this “sensor blind time” to about 1 s is possible using ceramic hot plates as originally designed in [42] and demonstrated in [43]. Applying the sensors on top of silicon micro hotplates as described for instance in [41,44–46], the sensor blind time can be reduced to below 100 ms. After regeneration, the next measurement period follows. Data are delivered until the saturation effects become noticeable (*i.e.*, at about 30% of the maximum resistance change). At that point, the next regeneration is initiated.

It is a specific feature of this setup that the measurement range can be self-adapted with this concept. If the saturation level is reached very soon after the last regeneration, this is a strong indication for a high mean NO_x_ concentration requiring an extended measurement range. Then, after the following regeneration period, the sensor may be cooled to a lower operation temperature which reduces the sensor’s sensitivity as illustrated in Figure 8(d), and *R*_0_ is set to the new value. In other words, the measurement range can be self-adapted—without losing linearity—taking advantage of the temperature dependency of the sensitivity.

It is noteworthy to mention that all classical metal oxide sensors have a temperature dependence of the zero-NO_x_ offset that will severely affect their accuracy, especially when low NO_x_ concentration shall be measured. The integrating sensor does too, but in the instantaneous mode (via the derivative) the NO_x_ offset is zero per definition. The sensitivity changes with temperature, but not with the instantaneous NO_x_ offset. This is unique for a resistive or impedimetric sensor.

### 4.6. Selectivity and Cross-Sensitivities to Other Gas Components

The accuracy of a gas sensor is often restricted by its selectivity to the analyte. In the lean automotive exhaust as well as in the ambient air, the gas components will vary (in particular O_2_, CO_2_ and H_2_O concentrations), necessitating the study of their influences on the sensor signal.

The sensitivity of our integrating NO_x_ sensor to NO_x_ is based on the carbonate-nitrate conversion. These reactions [including NO oxidation and NO_2_ storage according to Equations (3) and (2)] are influenced by the concentrations of O_2_ and CO_2_. In addition to the influence of CO_2_, H_2_O was also found to affect the catalytic performance of a K-based LNT [47]. Therefore, it is expected that variations in all three gas components might lead to cross-sensitivities. Initial studies on the influence of CO_2_ and O_2_ on the response of the integrating NO_x_ sensor were performed [15]:

- The presence of oxygen is essential for the accumulation of NO_x_ in the sensitive layer, but variation from 2.5% to 7.5% O_2_ was found to have no influence on the sensor signal.
- CO_2_ variations were also found to be negligible in the concentration range from 1.5% to 10%.

The effect of H_2_O on the sensing characteristics has not been investigated yet. However, it is clear that the automotive exhaust components O_2_, H_2_O, and CO_2_ do not vary independently. They depend on the air-to-fuel ratio, in other words an increasing O_2_ concentration comes along with a decreasing CO_2_ and H_2_O concentration. Therefore, the sensor signal dependency on the air-to-fuel ratio will be subject of further studies.

LNTs are known to suffer from sulfur poisoning [34]: SO_2_, which origins from the sulfur content in diesel fuel, competes with NO_2_ for the storage sites, forming highly stable sulfates and leading to the deactivation of the storage capacity. Additionally, sulfur oxides occupy the catalytically active precious metal sites, lowering the oxidizing properties of the catalyst. Therefore, BaCO_3_ can be applied successfully as a sulfur adsorber layer to prevent thick film exhaust gas sensors from sulfation [48]. Toops *et al.* [49] report that the temperature dependent SO_2_ deactivation of a K-based LNT is mainly attributed to a loss in active storage sites. This loss in storage capacity after exposure to SO_2_ is expected to lower the sensitivity and measurement range of the integrating NO_x_ sensor. The effect of SO_2_ on the sensing performance is currently under investigation.

## 5. Conclusions

The integrating sensing principle is suited for the reliable detection of low concentrations of analyte. Besides the dose (or the amount), the actual analyte concentration can be determined by calculating the signal derivative, offering a second functionality for the sensor. We present an accumulative-type impedimetric NO_x_ sensor based on a NO_x_ storage catalyst material as sensitive layer. The sensor can be operated at temperatures from 300 to 400 °C which is suited for different applications requiring mean value detection like air quality monitoring or OBD in the automotive exhaust. The sensitivity is neither dependent on the actual NO_x_ concentration nor on the NO/NO_2_ ratio. Another benefit of this sensor compared to many concentration-detecting semiconducting devices is that the sensor responds and recovers fast. The determined response times or recovery times of 5 to 7 s are in the range of the gas exchange time of the test setup. Due to the accumulating properties, even small levels like 1,340 ppm·s or 200 ppb NO can be monitored time-continuously with a sufficient accuracy. The periodic regeneration of the storage sites ensures linearity, avoids a long-term base-line drift of the sensor signal via adjusting the zero value and enables a self-adaption of the linear measurement range taking advantage of the temperature dependency of the sensitivity.

## References

[1] Sberveglieri G. (1995). Recent developments in semiconducting thin-film gas sensors. Sens. Actuat. B.

[2] Moos R., Sahner K., Fleischer M., Guth U., Barsan N., Weimar U. (2009). Solid state gas sensor research in Germany—A status report. Sensors.

[3] Korotcenkov G. (2007). Metal oxides for solid-state gas sensors: What determines our choice?. Mater. Sci. Eng. B.

[4] Barsan N., Weimar U. (2003). Understanding the fundamental principles of metal oxide based gas sensors; The example of CO sensing with SnO_2_ sensing in the presence of humidity. J. Phys. Condens. Matter.

[5] Gouma P.I., Kalyanasundaram K. (2008). A selective nanosensing probe for nitric oxide. Appl. Phys. Lett.

[6] Menil F., Coillard V., Lucat C. (2000). Critical review of nitrogen monoxide sensors for exhaust gases of lean burn engines. Sens. Actuat. B.

[7] Kannan S., Steinebach H., Rieth L., Solzbacher F. (2010). Selectivity, stability and repeatability of In_2_O_3_ thin films towards NO_x_ at high temperatures (≥500 °C). Sens. Actuat. B.

[8] Sasaki H., Scholl D., Parsons M., Hiroshi I., Shiotani K., Visser J., Zawacki G., Kawai T., Teramoto S., Kubinski D. (2010). Development of an Al_2_O_3_/ZrO_2_-composite high-accuracy NO_x_ sensor. SAE Tech. Paper.

[9] (2008). Directive 2008/50/EC of the European Parliament and of the Council of 21 May 2008 on Ambient Air Quality and Cleaner Air for Europe. Off. J. EU.

[10] (2010). BImSchV. Neununddreißigste Verordnung zur Durchführung des Bundes-Immissionsschutz-gesetzes (Verordnung über Luftqualitätsstandards und Emissionshöchstmengen—39. BImSchV). Bundesgesetzblatt Jahrgang 2010.

[11] Haugen J.-E., Tomic O., Kvaal K. (2000). A calibration method for handling the temporal drift of solid state gas-sensors. Anal. Chim. Acta.

[12] Capone S., Epifani M., Francioso L., Kaciulis S., Mezzi A., Siciliano P., Taurino A.M. (2006). Influence of electrodes ageing on the properties of the gas sensors based on SnO_2_. Sens. Actuat. B.

[13] Cantalini C., Wlodarski W., Sun H.T., Atashbar M.Z., Passacantando M., Santucci S. (2000). NO_2_ response of In_2_O_3_ thin film gas sensors prepared by sol–gel and vacuum thermal evaporation techniques. Sens. Actuat. B.

[14] Yuan L., Hyodo T., Shimizu Y., Egashira M. (2011). Preparation of mesoporous and/or macroporous SnO_2_-based powders and their gas-sensing properties as thick film sensors. Sensors.

[15] Geupel A., Schönauer D., Röder-Roith U., Kubinski D.J., Mulla S., Ballinger T.H., Chen H.-Y., Visser J.H., Moos R. (2010). Integrating nitrogen oxide sensor: A novel concept for measuring low concentrations in the exhaust gas. Sens. Actuat. B.

[16] Geupel A., Kubinski D.J., Mulla S., Ballinger T.H., Chen H.-Y., Visser J.H., Moos R. (2011). Integrating NO_x_ sensor for automotive exhausts—A novel concept. Sens. Lett.

[17] Beulertz G., Groß A., Moos R., Kubinski D.J., Visser J.H. (2012). Determining the total amount of NOx in a gas stream—Advances in the accumulating gas sensor principle. Sens. Actuat. B.

[18] Moos R., Wedemann M., Spörl M., Reiß S., Fischerauer G. (2009). Direct catalyst monitoring by electrical means: An overview on promising novel principles. Top. Catal.

[19] Shu J.H., Wikle H.C., Chin B.A. (2010). Passive chemiresistor sensor based on iron (II) phthalocyanine thin films for monitoring of nitrogen dioxide. Sens. Actuat. B.

[20] Li J., Lu Y., Ye Q., Cinke M., Han J., Meyyappan M. (2003). Carbon nanotube sensors for gas and organic vapor detection. Nano Lett.

[21] Moos R. (2010). Catalysts as sensors—A promising novel approach in automotive exhaust gas aftertreatment. Sensors.

[22] Rodriguez-Gonzalez L., Rodriguez-Castellon E., Jimenez-Lopez A., Simon U. (2008). Correlation of TPD and impedance measurements on the desorption of NH_3_ from zeolite H-ZSM-5. Solid State Ion.

[23] Rodriguez-Gonzalez L., Simon U. (2010). NH_3_-TPD measurements using a zeolite-based sensor. Meas. Sci. Technol.

[24] Matsuguchi M., Kadowaki Y., Tanaka M. (2005). A QCM-based NO_2_ gas detector using morpholine-functional cross-linked copolymer coatings. Sens. Actuat. B.

[25] Jung W., Sahner K., Leung A., Tuller H.L. (2009). Acoustic wave-based NO_2_ sensor: Ink-jet printed active layer. Sens. Actuat. B.

[26] Maruo Y.Y., Kunioka T., Akaoka K., Nakamura J. (2009). Development and evaluation of ozone detection paper. Sens. Actuat. B.

[27] Small W., Maitland D.J., Wilson T.S., Bearinger J.P., Letts S.A., Trebes J.E. (2009). Development of a prototype optical hydrogen gas sensor using a getter-doped polymer transducer for monitoring cumulative exposure: Preliminary results. Sens. Actuat. B.

[28] Kubinski D.J., Visser J.H. (2008). Sensor and method for determining the ammonia loading of a zeolite SCR catalyst. Sens. Actuat. B.

[29] Varshney C.K., Singh A.P. (2003). Passive samplers for NO_x_ monitoring: A critical review. Environmentalist.

[30] Helwig A., Müller G., Garrido J.A., Eickhoff M. (2008). Gas sensing properties of hydrogen-terminated diamond. Sens. Actuat. B.

[31] Fremerey R., Reiß S., Geupel A., Fischerauer G., Moos R. (2011). Determining of the NO_x_ loading of an automotive lean NO_x_ trap by directly monitoring of the electrical properties of the catalyst material itself. Sensors.

[32] Roy S., Baiker A. (2009). NO_x_ Storage catalysis: From mechanism und materials properties to storage-reduction performance. Chem. Rev.

[33] Takeuchi M., Matsumoto S. (2004). NO_x_ storage-reduction catalysts for gasoline engines. Top. Catal.

[34] Epling W.S., Campbell L.E., Yezerets A., Currier N.W., Parks J.E. (2004). Overview of the fundamental reactions and degradation mechanism of NO_x_ storage/reduction catalysts. Catal. Rev.

[35] Lesage T., Saussey J., Malo S., Hervieu M., Hedouin C., Blanchard G., Daturi M. (2007). Operando FTIR study of NO_x_ storage over a Pt/K/Mn/Al_2_O_3_-CeO_2_ catalyst. Appl. Catal. B.

[36] Dawody J., Skoglundh M., Wall S., Fridell E. (2005). Role of Pt-precursor on the performance of Pt/BaCO_3_/Al2O_3_·NO_x_ storage catalysts. J. Mol. Catal. A.

[37] Nova I., Castoldi L., Lietti L., Tronconi E., Forzatti P., Prinetto F., Ghiotti G. (2004). NO_x_ adsorption study over Pt-Ba/alumina catalysts: FT-IR and pulse experiments. J. Catal.

[38] Chen H.-Y., Mulla S., Ballinger T.H. (2010). NO_x_ storage Materials for Sensor Applications.

[39] Schütze A., Pieper N., Zacheja J. (1995). Quantitative ozone measurement using a phthalocyanine thin-film sensor and dynamic signal evaluation. Sens. Actuat. B.

[40] Forzatti P., Castoldi L., Nova I., Lietti L., Tronconi E. (2006). NO_x_ removal catalysis under lean conditions. Catal. Today.

[41] Simon I., Barsan N., Bauer M., Weimar U. (2001). Micromachined metal oxide gas sensors: Opportunities to improve sensor performance. Sens. Actuat. B.

[42] Rettig F., Moos R. (2004). Ceramic meso hot-plates for gas sensors. Sens. Actuat. B.

[43] Kita J., Rettig F., Moos R., Drüe K.-H., Thust H. (2005). Hot-plate gas sensors—Are ceramics better?. Int. J. Appl. Ceram. Technol.

[44] Faglia G., Comini E., Cristalli A., Sberveglieri G., Dori L. (1999). Very low power consumption micromachined term CO sensors. Sens. Actuat. B.

[45] Semancik S., Cavicchia R.E., Wheeler M.C., Tiffany J.E., Poirier G.E., Walton R.M., Suehle J.S., Panchapakesan B., DeVoe D.L. (2001). Microhotplate platforms for chemical sensor research. Sens. Actuat. B.

[46] Friedberger A., Kreisl P., Rose E., Müller G., Kühner G., Wöllenstein J., Böttner H. (2003). Micromechanical fabrication of robust low-power metal oxide gas sensors. Sens. Actuat. B.

[47] Toops T.J., Smith D.B., Epling W.S., Parks J.E., Partridge W.P. (2005). Quantified NO_x_ adsorption on Pt/K/gamma-Al_2_O_3_ and the effects of CO_2_ and H_2_O. Appl. Catal. B.

[48] Rettig F., Moos R., Plog C. (2003). Sulfur adsorber for thick-film exhaust gas sensors. Sens. Actuat. B.

[49] Toops T.J., Smith Pihl J.A. (2008). Sulfation of potassium-based lean NO_x_ trap while cycling between lean and rich conditions: I. Microreactor study. Catal. Today.

